# Machine Learning-Based Evaluation of Shear Capacity of Recycled Aggregate Concrete Beams

**DOI:** 10.3390/ma13204552

**Published:** 2020-10-13

**Authors:** Yong Yu, Xinyu Zhao, Jinjun Xu, Cheng Chen, Simret Tesfaye Deresa, Jintuan Zhang

**Affiliations:** 1 School of Environment and Civil Engineering, Dongguan University of Technology, Dongguan 523808, China; yuyong1990@foxmail.com; 2 School of Aerospace Engineering, Xi’an Jiaotong University, Xi’an 710049, China; 3 State Key Laboratory of Subtropical Building Science, South China University of Technology, Guangzhou 510640, China; 4 College of Civil Engineering, Nanjing Tech University, Nanjing 211816, China; simtes2009@gmail.com; 5 State Key Laboratory of Green Building in Western China, Xi’an University of Architecture and Technology, Xi’an 710055, China; 6 Hunan Engineering Technology Research Center for High Speed Railway Operation Safety Assurance, Hunan Vocational College of Railway Technology, Zhuzhou 412001, China; chencheng401@sina.com; 7 College of Architecture and Electrical Engineering, Hezhou University, Hezhou 542899, China; zhangjthzu@163.com

**Keywords:** recycled aggregate concrete, beam, shear capacity, Grey relational analysis, machine learning

## Abstract

Recycled aggregate concrete (RAC) is a promising solution to address the challenges raised by concrete production. However, the current lack of pertinent design rules has led to a hesitance to accept structural members made with RAC. It would entail even more difficulties when facing application scenarios where brittle failure is possible (e.g., beam in shear). In this paper, existing major shear design formulae established primarily for conventional concrete beams were assessed for RAC beams. Results showed that when applied to the shear test database compiled for RAC beams, those formulae provided only inaccurate estimations with surprisingly large scatter. To cope with this bias, machine learning (ML) techniques deemed as potential alternative predictors were resorted to. First, a Grey Relational Analysis (GRA) was carried out to rank the importance of the parameters that would affect the shear capacity of RAC beams. Then, two contemporary ML approaches, namely, the artificial neural network (ANN) and the random forest (RF), were leveraged to simulate the beams’ shear strength. It was found that both models produced even better predictions than the evaluated formulae. With this superiority, a parametric study was undertaken to observe the trends of how the parameters played roles in influencing the shear resistance of RAC beams. The findings indicated that, though less influential than the structural parameters such as shear span ratio, the effect of the replacement ratio of recycled aggregate (RA) was still significant. Nevertheless, the value of *v*_c_/(*f*_c_)^1/2^ (i.e., the shear contribution from RAC normalized with respect to the square root of its strength) predicted by the ML-based approaches appeared to be insignificantly affected by the replacement level. Given the existing inevitable large experimental scatter, more shear tests are certainly needed and, for safe application of RAC, using partial factors calibrated to consider the uncertainty is feasible when designing the shear strength of RAC beams. Some suggestions for future works are also given at the end of this paper.

## 1. Introduction

Mountains of concrete waste are being generated from demolition sites. This fact, together with the exceptional recent growth of concrete consumption, puts the construction sector into a dilemma. Such a situation, though, in turn, urges the sector to facilitate the development of strategies to mitigate concrete’s negative impacts [1]. Recycling concrete waste is one such attempt, in which recycled aggregate (RA) crushed from concrete rubble is used to replace natural aggregate (NA) needed in new concrete. The application of the resulting products, namely, the recycled aggregate concrete (RAC), is attracting immense interest, and believed to be able to open up new vistas for sustainability [2,3].

Despite such benefits, the application of RAC in structures is still not widespread. This is caused not only by the simple reluctance to the change of common practice, but also by many legitimate concerns. One of the biggest concerns is the inferior properties of RA, which would result in significant reductions in the mechanical properties of RAC [4,5,6,7]. This should impede the acceptance of RAC-based members, though many laboratory studies have revealed their structural feasibility [8,9,10,11,12,13,14]. Another wave of concern is around design and modeling, where the validity of previous code-based models and analytical procedures developed for natural aggregate concrete (NAC) components is questioned for their RAC counterparts; this issue has begun to be systematically addressed [15,16,17].

When it comes to RAC beams, the shear performance of the components is of particular concern. This is simply because the shear failure is generally sudden and often catastrophic. Moreover, there are additional worries that are quite understandable: owing to the inherent porosity and heterogeneity of recycled aggregates, the response of a RAC beam in shear would differ markedly from that of a NAC beam—the former is likely to exhibit weaker aggregate interlock action, smoother shear crack planes, and consequently, lower shear capacity and less ductile behavior [1].

Up to now, significant efforts have been made worldwide in response to the doubts just mentioned [18,19,20,21,22,23,24,25,26,27,28,29,30,31,32,33,34,35,36,37,38]. Early shear tests [18,19,20] indicated that RAC beams have similar cracking pattern and failure modes with those of NAC ones, but present lower diagonal cracking load and inferior shear-resisting capacity. From then on, interest has been sparked, providing more invaluable experimental evidence into this area [21,22,23,24,25,26,27,28,29,30,31,32,33,34,35,36,37,38]. For example, Fathifazl et al. [24], Arezoumandi et al. [25,26], and Knaack and Kurama [27] investigated the effects of replacement level and longitudinal reinforcement ratio on the shear behavior of RAC beams. The results showed that an increased amount of RA may erode the beams’ initial stiffness, but the effect on the shear strength is relatively small; given the same volume of longitudinal reinforcement, the shear capacity of RAC beams is clearly lower than that of NAC beams [25]. Comparisons were also made between RAC beams with and without shear reinforcement [32,33]. An overall trend observed is that for shear-reinforced beams, a great portion of resistance is contributed by web steel, and there is no such evidence that the resistance is significantly affected by the use of recycled aggregates. Hence, in such cases (with shear reinforcement), the detrimental effects are not as prominent as in beams without shear reinforcement. Tošić et al. [39] added their own test data to a comprehensive database for RAC beams, and evaluated the beams’ flexural and shear strengths using European design code. In the shear loading case, the code can successfully predict the ultimate force of RAC beams without stirrups, but it fails for the beams with stirrups. In this regard, much more investigations for RAC beams with web steel are required. This is not surprising, since a recent study by Pacheco et al. [40] has confirmed that, after a strict filtering of previous test data, only eighteen shear-reinforced RAC beams are considered suitable for the examination of code provisions, indicating the very scarcity of such tests. It also turned out that the code-based predictions for RAC beams with stirrups are more conservative and show higher variability than those for RAC beams without stirrups [40]. Such a finding seemingly contradicts one’s initial intuition, but just highlights the complex nature of RAC beams in shear.

Thus, the shear problem of RAC beams has not been fundamentally and conclusively solved yet. This is particularly true given the intrinsic uncertainties involved—the amount of residual mortar attached to RA is usually unknown (the main reason why recycled aggregate is more porous and heterogeneous than natural aggregate), nor the RA source. Furthermore, limited understanding of the shear failure mechanism per se (even for the case of NAC) adds to the complexity, which encourages further explorations for properly representing the beams’ shear behavior.

From another angle, it is noticed that machine learning (ML) techniques are being increasingly used for complex material and structural problems [41,42,43]. Those problems often defy solutions via traditional approaches but can get satisfactory answers by ML techniques. In these techniques, an intelligent mathematical system is first trained by feeding a set of examples (data and answers), with the aim of minimizing the difference between outputs and answers. By so doing, the system with a predefined algorithm is enabled to come up with rules that can eventually deal with new input data and give accurate predictive outputs. At the core of ML techniques is the transformation of the data (inputs) into meaningful representations of hidden mechanisms, which is otherwise a grueling challenge to conventional theoretical approaches.

The widely accepted ML approaches include, among many others, artificial neural network, kernel methods like support vector machine, and decision trees like random forest [41,42,43]. These models have been effectively used as predictive modeling approaches for normal concrete (e.g., [44]) as well as recycled concrete [45,46,47,48].

This paper intends to quantify the shear capacity of RAC beams by means of machine learning techniques. To this end, a comprehensive experimental database containing 264 beam specimens was first constructed. Then, nine shear design methods collected from both code standards and scientific publications were used to examine their effectiveness for the case of RAC shear beams. After this appraisal, a Grey relational analysis (GRA) was carried out to rank the importance of potential parameters that would affect the beams’ shear resistance. With this requisite information, two ML approaches—the artificial neural network (ANN) and the random forest (RF)—were developed to evaluate the shear capacity of RAC beams. Once trained and tested successfully, the two approaches were finally utilized to conduct a parametric study, by which the overall trend can be well observed of how the shear capacity of RAC beams is influenced by the selected parameters. This study helps to understand the shear problem of RAC beams, and hence contributes to upscaling the use of RAC in structural applications with confidence.

## 2. Shear Test Database and Existing Prediction Methods

### 2.1. Experimental Database

A comprehensive experimental database of RAC beams loaded in shear and their reference beams made with NAC was constructed in this study, as detailed in Appendix A Table A1. This database comprised 264 datasets (206 beams without steel stirrups and 58 beams containing steel stirrups) collected from 28 published works across the world [18,19,20,21,22,23,24,25,26,27,29,30,31,32,33,34,35,36,37,38,49,50,51,52,53,54,55,56]. Experimental outcomes published in Chinese [49,50,51,52,53,54,55,56] were also included as a complement. When constructing the test database, the following criteria were applied:

1. Only beams experiencing shear failure were considered. Data related to flexural failure were excluded. Note that in some test beams more stirrups were used in one shear span to control the shear failure to occur in another span. For these cases, the maximum load recorded in the less shear reinforced span was taken as the ultimate shear resistance of the specimen;
2. Key mix proportioning factors of RAC, especially the replacement ratio and the water-to-cement ratio, must be reported; otherwise, the specimens were not included;
3. Beams incorporating waste materials other than the recycled coarse aggregate (e.g., bricks, recycled fine aggregate) or fibers were not included; beams reinforced with FRP bars were also not considered;
4. Finally, datasets with inadequate information for the construction of Appendix A Table A1 were removed.

Note that in the previous study by Pacheco et al. [40] beams with a shear span ratio less than 2.5 were excluded for properly evaluating the applicability of existing shear design provisions for RAC beams. Additionally, Pacheco et al. [40] found that a high longitudinal reinforcement ratio (approaching 4.0%) may influence the code validation. In this study, these test data were retained because we intend to develop a unified ML-based predictive approach that adapts to various parameters with a wide range. Still, when checking the appropriateness of shear design methods (see the next section), the scope of each method will be strictly followed.

All of the specimens reported in Appendix A Table A1 were the simply supported rectangular beams subjected to four-point loading. The table gives the following fundamental attributes for each specimen: (1) their geometry characteristics (section width *b*, section height *h*, effective section height *d*, and shear span ratio *a*/*d*); (2) replacement ratio of RA *r*; (3) water-to-cement ratio *w*/*c*; (4) maximum size of RA pieces *s*_max_; (5) compressive strength of concrete *f*_c_; (6) longitudinal and transverse reinforcement ratios, *ρ*_s_ and *ρ*_sv_, respectively; (7) yield strengths of the longitudinal and transverse reinforcement, *f*_y_ and *f*_yv_, respectively; and (8) experimentally obtained ultimate shear force *V*_u_. In this paper, the normalized ultimate shear stress (i.e., *v*_u_ = *V*_u_/*bd* [57,58]) is used as a metric for evaluating the beam’s shear resistance.

The shear resisting mechanism in RAC beams is controlled by a set of parameters, which can be summarized as:

- The compressive strength of concrete, *f*_c_, is typically considered the most relevant factor to the shear capacity;
- The replacement ratio of RA, *r*, is a unique factor for beams made with RAC, and would affect the magnitude and variability of *f*_c_;
- The maximum size of RA, *s*_max_, influences the interlock action at shear cracking planes and hence the ultimate shear resistance;
- The shear span ratio, *a*/*d*, and the beam section height, *h*, play vital roles in determining the shear failure mode of beams and the size effect on shear strength, respectively [59,60].
- The longitudinal reinforcement ratio, *ρ*_s_, directly determines the dowel effect which constitutes an important and integral part of the shear resisting mechanism;
- The parameter, *ρ*_sv_*f_yv_*, represents the essential contribution from transverse reinforcement.

Table 1 shows the statistics of the above parameters (together with the ultimate shear stress *v*_u_). Those parameters have relatively wide ranges, while their average values reflect common engineering practice. The mean concrete strength (32.7 MPa), for instance, is a normal value commonly applied in beams, the mean shear span ratio (2.71) falls within the range associated with shear-dominated failure, and the mean longitudinal reinforcement ratio (0.0181) also represents ordinary flexural reinforcing schemes for beams.

### 2.2. Shear Capacity Assessment with Existing Methods

In this section, nine classic shear design methods presented in existing codes or publications were used to evaluate the shear strength of beams in the test database. Those methods, as tabulated in Appendix A Table A2, correspond to the provisions in the ACI Building Code (ACI318-2014) [61], the British Standard EN1992-1-1:2004 [62], the Chinese Concrete Code (GB50008-2010) [63], the Japanese Code (JSCE Guideline No. 15) [64], and the New Zealand Standard (NZS3101-2006) [65]. The Zsutty’s early contribution [57,58], the simplified modified compression field theory [66,67] (note that the simplified MCFT theory has been adopted in the CEB-FIP Model Code 2010 [68] and the Canada Code CSA A23.3-19 [69]), the critical shear crack theory [70,71] and the Zhang’s plasticity theory [72] were also chosen for the assessment. These nine methods have either been accredited by national standard committees or well accepted by the professional community.

**Figure 1:**
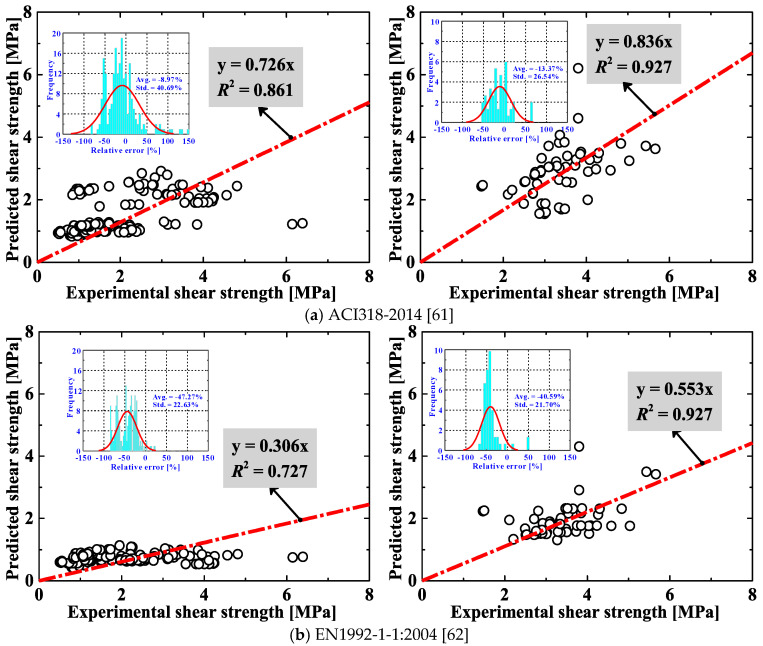

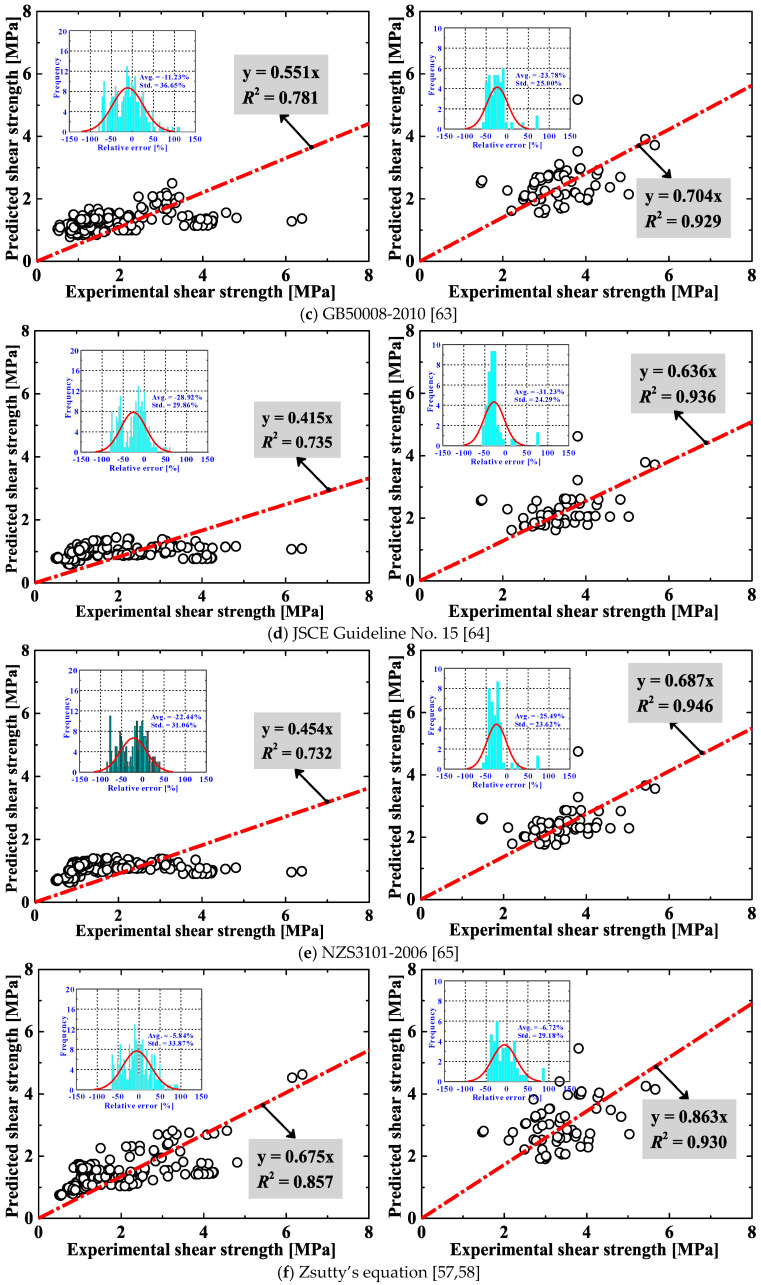

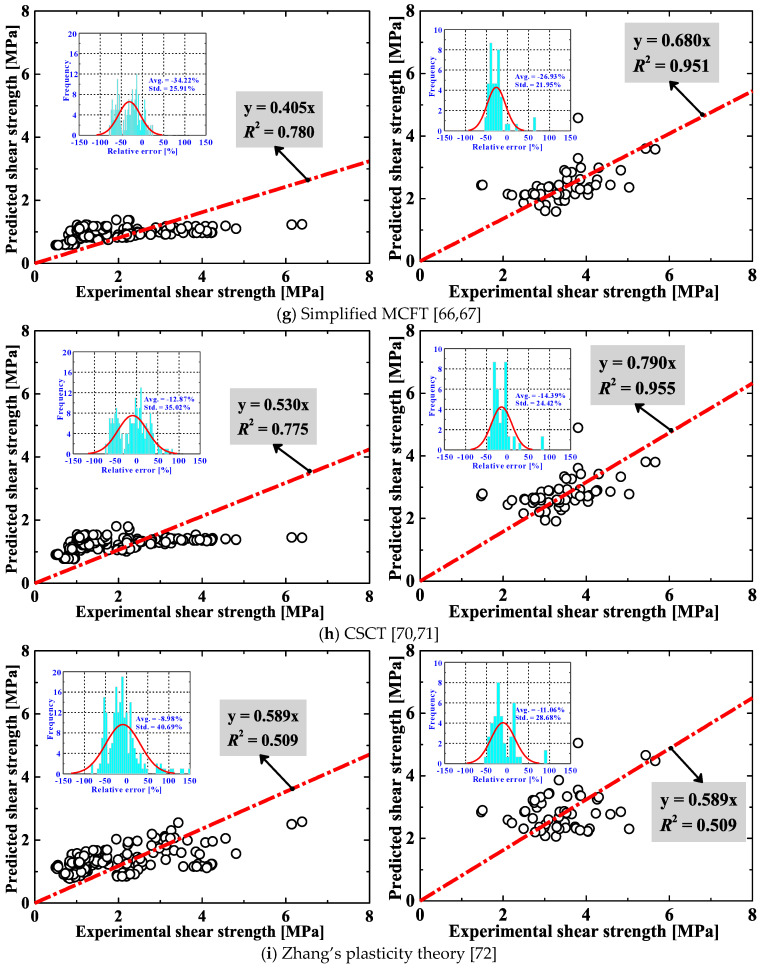
Comparison between predicted and experimental shear strengths for different methods (Left column: beams without stirrups; Right column: beams with stirrups).

Comparisons between the experimental and the predicted shear strengths for RAC beams without/with steel stirrups are, respectively, shown in the left and right columns of Figure 1. As seen in the figure, the predicted-to-experimental ratio varies from one method to another. For beams without stirrups, those ratios are located within the range of 0.306 to 0.726, indicating a poor predictive performance for all of the shear design methods. As stirrups were incorporated, the results improve to some extent (the predicted-to-experimental ratio ranges from 0.553 to 0.836). This is probably because the presence of transverse reinforcement which participates in the shear resisting mechanism tends to reduce the experimental brittleness (and hence increase the prediction accuracy) [22]. The outputs in both cases (without and with stirrups) suggest an inaccuracy and over-conservatism for all the evaluated methods. Moreover, for beams without stirrups, a high scatter is exhibited for all the methods (their *R*^2^ are all below 0.8 except the ACI318-2014 method [61] and Zsutty’s equation [57,58]). Combining the prediction results for RAC beams without/with stirrups, the best performance goes to the Zsutty’s equation where its average value of *R*^2^ is equal to 0.883. Overall, the above results indicate that the predictions of these existing methods are generally unsatisfactory (i.e., showing high variability), especially for RAC beams without steel stirrups.

The major contributing factors to the inadequacy of the evaluated methods may include: (1) the existing methods are established primarily for conventional concrete beams, and thus cannot convey the particular material characteristics of RAC; (2) due to the brittleness feature of shear failure, judicious and prudent considerations must be paid when developing shear equations [73,74,75]. This inevitably leads to a conservatism (more or less) for concrete beams in shear, irrespective of their concrete type; and (3) the detrimental impact of incorporating RA becomes more obvious with increasing concrete strength [40], consequently posing significant safety challenges when applying existing methods to these cases without any modification. As such, establishing an accurate shear resistance prediction for RAC beams is indeed a strenuous task and necessitates more effort.

## 3. Parametric Importance Evaluation Using Grey Relational Analysis

### 3.1. Principle of Grey Relational Analysis

Prior to conducting the ML-based analysis, it is useful to identify the importance of various parameters. Such a rehearsal can provide insights into whether the selected input parameters are appropriate [12,13]. To achieve this objective, the powerful mathematical tool of Grey relational analysis (GRA), which has been proposed to deal with poor, incomplete, and uncertain systems [76], was used in this study.

Conceptually, the knowledge about a system is composed of white information (have been well understood), black information (have not been grasped due to difficulties such as epistemic uncertainty) and grey information (somewhere in between the white and black information). Although the relationships between sequences (parameters and targets) are not clear, the grey information exists in reality [76]. Nicely, the GRA is such a method that can capture those relationships between sequences with grey information. It is particularly suitable for solving problems with complex interrelationships between multiple variables, thus facilitating the developers to discern the relative importance of candidate parameters.

In the present GRA work, the ultimate shear stress *v*_u_ of beam specimens in Appendix A Table A1 was defined as the benchmark matrix *X*_0_. The possible influential parameters, including the compressive strength of concrete *f*_c_, the RA replacement ratio *r*, the maximum aggregate size of RA *s*_max_, the geometric characteristics of RAC beams (i.e., *h* and *a*/*d*), and the reinforcement properties (*ρ*_s_ and *ρ*_sv_*f_yv_*) were selected as the derivative matrix *X_i_*. The following formulae relating the benchmark matrix *X*_0_ and the derivative matrix *X_i_* were employed to derive the grey relational factor *γ*_0_*_i_*, which can be used as indicators to represent the parametric importance:

(1) $$\gamma_{0i}=\gamma \left(X_{0},X_{i}\right)=\frac{1}{n}\sum_{w=1}^{n}\gamma_{0i}\left(w\right)$$

(2) $$\begin{array}{l} \gamma_{0i}\left(w\right)=\gamma \left(x_{0}(w),x_{i}(w)\right)\\ \;\;\;\;\;\;\;\;\;\;=\frac{\underset{i}{\min}\;\underset{w}{\min}\left|x_{0}(w)-x_{i}(w)\right|+\xi \underset{i}{\max}\;\underset{w}{\max}\left|x_{0}(w)-x_{i}(w)\right|}{\left|x_{0}(w)-x_{i}(w)\right|+\xi \underset{i}{\max}\;\underset{w}{\max}\left|x_{0}(w)-x_{i}(w)\right|} \end{array}$$

(3) $$\left(\begin{array}{l} X_{0}\\ X_{1}\\ \ldots \\ X_{i}\\ \ldots \\ X_{m} \end{array}\right)=\left(\begin{array}{l} x_{0}(1),\;x_{0}(2),\;\ldots \;,\;x_{0}(n)\\ x_{1}(1),\;x_{1}(2),\;\ldots \;,\;x_{1}(n)\\ \;\;\;\;\;\;\;\;\;\;\;\;\;\;\ldots \\ x_{i}(1),\;x_{i}(2),\;\ldots \;,\;x_{i}(n)\\ \;\;\;\;\;\;\;\;\;\;\;\;\;\;\ldots \\ x_{m}(1),\;x_{m}(2),\;\ldots \;,\;x_{m}(n) \end{array}\right)$$

where *ξ* is a constant, and its value ranges from 0 to 1.0. Normally, *ξ* can be set as 0.5 [76].

The range of the Grey relational factor *γ*_0_*_i_*, is between 0 and 100%. It is noted that a value of *γ*_0_*_i_* approaching 100% represents a very tight correlation between sequences; if *γ*_0_*_i_* is close to 70%, there is still a strong correlation; if *γ*_0_*_i_* is less than 50%, the correlation can be regarded as loose to negligible [76].

### 3.2. Results of Parametric Importance

**Figure 2:**
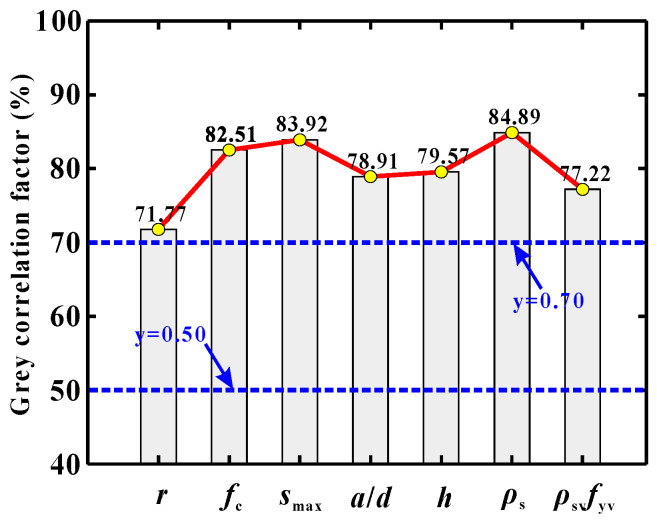
Parametric importance evaluated by Grey relational analysis (GRA).

Figure 2 shows the calculation results for the Grey relational factor. From the figure, the order of the parametric importance is readily identified as: *ρ_s_* > *s*_max_ > *f*_c_ > *h* > *a*/*d* > *ρ_sv_f_yv_* > *r*.

Clearly, the three parameters *ρ*_s_, *s*_max_ and *f*_c_ have the greatest impacts on the shear resistance (their Grey relational factors are 84.89%, 83.92% and 82.51%, respectively). Next, the parameters *h*, *a*/*d* and *ρ_sv_f_yv_* also have considerable effects on the load-carrying capacity (their Grey relational factors are all below 80% but higher than 75%). Last but not least, the GRA results indicate that the influence of the RA replacement level, *r*, cannot be overlooked, since its Grey relational factor is over 70% (= 71.77%). In view of the above results, all of the seven parameters were chosen as the input parameters in the following ML modeling.

A note worthy of mentioning is that the replacement level, *r*, has been understood to influence the compressive strength, *f*_c_, of RAC. Thus, one may suspect that the parameter *r* should not be considered in the ML modeling. However, it is also believed that using *f*_c_ alone is incapable of reflecting the material variability of RAC but *r* does (at least to some extent). Moreover, the Grey relational analysis, which has an extraordinary capacity of quantifying the correlative extent of sequences, has identified the particular importance of *r*. Thus *r* was incorporated in conjunction with *f*_c_ in the ML analysis, in which the dependence of *f*_c_ on *r* can be automatically determined by adjusting the internal relationships of the two parameters.

## 4. Shear Resistance Evaluation Using Machine Learning Approaches

In the present study, two Machine Learning approaches, i.e., the artificial neural network (ANN) and the random forest (RF), were employed to quantify the shear strength of RAC beams. The following are brief descriptions of their basic principles.

### 4.1. Artificial Neural Network

Among all ML techniques, the artificial neural network (ANN) is perhaps the most widely adopted model in simulating the structural or functional aspects of the human brain and nervous system [46,47]. This is due to the ANN’s high accuracy feature induced by the self-adjustment function for each layer’s weight.

**Figure 3:**
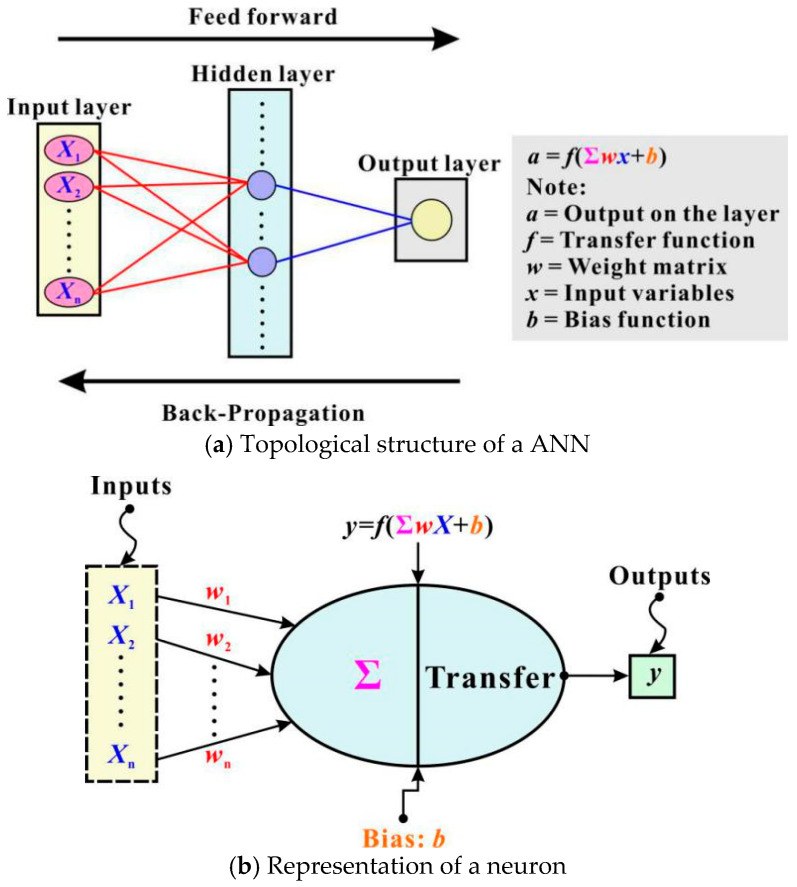
Schematic illustrations of artificial neural network (ANN) and neuron.

ANN is a powerful and versatile tool that can “learn” complex relationships between input and output. When “trained” with a prepared database, ANN can predict the result instantaneously, which makes it ideal for predicting the shear capacity of RAC beams. Figure 3a shows the classical layered structure for an ANN model. As seen there, a total of three categories of layers (namely, the input layer, the hidden layers and the output layer) exist. These layers are constituted by similar fundamental elements called neuron (see Figure 3b). Commonly, neurons between adjacent layers are highly-interconnected. They receive signals from the external environment or previous layer’s neurons, and then process and pass the signals to the next layer’s neurons. Through such a way, multiple layers of neurons with nonlinear transfer functions allow the network to predict complex nonlinear relationships between input and output.

As shown in Figure 3, neurons in the hidden layer(s) are connected to previous and next layers by network weights *w* and biases *b*. Generally, these neurons are responsible for executing two mathematical operations: (*i*) feed-forward and (*ii*) back-propagation. In a feed-forward operation, the first step is to weight the inputs and the second to sum the weighted inputs, which can be algebraically expressed as:

(4) $$net_{j}=\sum_{i=1}^{n}w_{ij}x_{i}+b_{j}$$

where *w*_ij_ is the connection weight relating the neuron *i* in the previous layer to the neuron *j* in the present layer; *x*_i_ is the output of the neuron *i*; *n* is the total amount of neutrons in the previous layer; *b*_j_ is the bias value; and *net*_j_ is the weighted sum (i.e., the collection of signals gathered at the neuron *j*).

Based on the weighted sum (*net*_j_), a transfer function *f*(out_j_) generating the outputs can be determined using a sigmoid function:

(5) $$f\left({\mathrm{out}}_{j}\right)=\frac{1}{1+e^{-net_{j}}}$$

It should be highlighted that the initially-generated outputs obtained from Equation (5) are unshaped and thus cannot be used to present the target performance due to the large random error. Therefore, in an ANN algorithm, the back-propagation operation is frequently used to reduce that error through adjusting the weights from the output layer back to the input layer until limiting the error to a sufficiently small range, which can be written as:

(6) $$\Delta w_{m}=\alpha \Delta w_{m-1}-\eta \frac{\partial E}{\partial w}$$

where *w* is the weight between any two neutrons; Δ*w*_n_ and Δ*w*_n-1_ denote the variations in the weight of *w* at *m* and *m*-1 iterations, respectively; *η* is the learning rate; *α* is the momentum coefficient. As described, the ANN model is trained by applying the feed-forward and back-propagation operations repeatedly in an iterative process. Once well trained, the ANN model can be used to predict new data.

To predict the RAC beam’s shear capacity, the ANN model developed in this study contained a total of seven input parameters (as described in the previous section) and one output parameter (i.e., the ultimate shear strength). Additionally, the neuron number of the hidden layer was determined as eight through multiple times of trial calculations.

Once developed, the numerical matrices of the input layer weights (IWs), the input layer biases (IBs), the hidden layer weights (HWs) and the hidden layer bias (HB) of the ANN can be finally fixed, as shown in Equations (7)–(10), respectively. They are extracted from the Matlab simulation environment [77], and can be directly used for predicting the beam’s shear capacity (see Equation (11)).

(7) $$\mathrm{IWs}=\left\{\begin{array}{ccccccc} -0.3993 & 0.6980 & -3.7035 & -0.5282 & 2.7461 & 0.6763 & -0.2151\\ -0.9876 & 2.3818 & -2.4138 & -4.0240 & 1.3073 & -3.1235 & -0.0118\\ 1.9092 & -0.7464 & -1.8895 & -1.3745 & -1.6888 & -3.1293 & -1.3166\\ 0.5613 & 0.2803 & -6.3300 & 2.6985 & -0.1238 & -0.9169 & -1.0698\\ 1.3680 & -1.0044 & -0.9299 & 0.5248 & 2.5228 & -0.7428 & 1.6202\\ 0.0963 & 2.2766 & 2.5643 & -2.5095 & -0.8605 & 1.0952 & 1.9664\\ 1.1415 & 0.0372 & 2.0690 & -1.6744 & -1.2017 & -3.8665 & 0.4589\\ 0.3155 & 0.9418 & 1.8594 & 2.8400 & -0.1145 & -4.9693 & 0.3829 \end{array}\right\}$$

(8) $${\mathrm{IBs}}^{T}=\{\begin{array}{cccccccc} 4.4300 & 0.1357 & 0.8424 & 0.9661 & 0.0302 & 2.1665 & 0.7274 & 4.5897 \end{array}\}$$

(9) $$\mathrm{HWs}=\left\{\begin{array}{cccccccc} 0.4199 & -0.0308 & 0.1082 & -0.2836 & -0.0263 & 0.4277 & -0.0424 & -1.0742 \end{array}\right\}$$

(10) HB={0.4011}

(11) $$y=\mathrm{HWs}\times g(\mathrm{IWs}\cdot x+\mathrm{IBs})+\mathrm{HB}\;\;\;\;\;\;\mathrm{where}\;g(x)=2/(1+e^{-2x})-1$$

### 4.2. Random Forest

Random forest (RF) is another effective ML approach aiming firstly to address the data storage problem [78]. In fact, RF is a modification of the decision tree method, which is characterized by a decision-making algorithm and its objective is to optimize the regression and classification procedure. Among many variants of the decision tree method, the regression decision tree (RDT) is representative, straightforward and easy-to-understand, as explained below.

**Figure 4:**
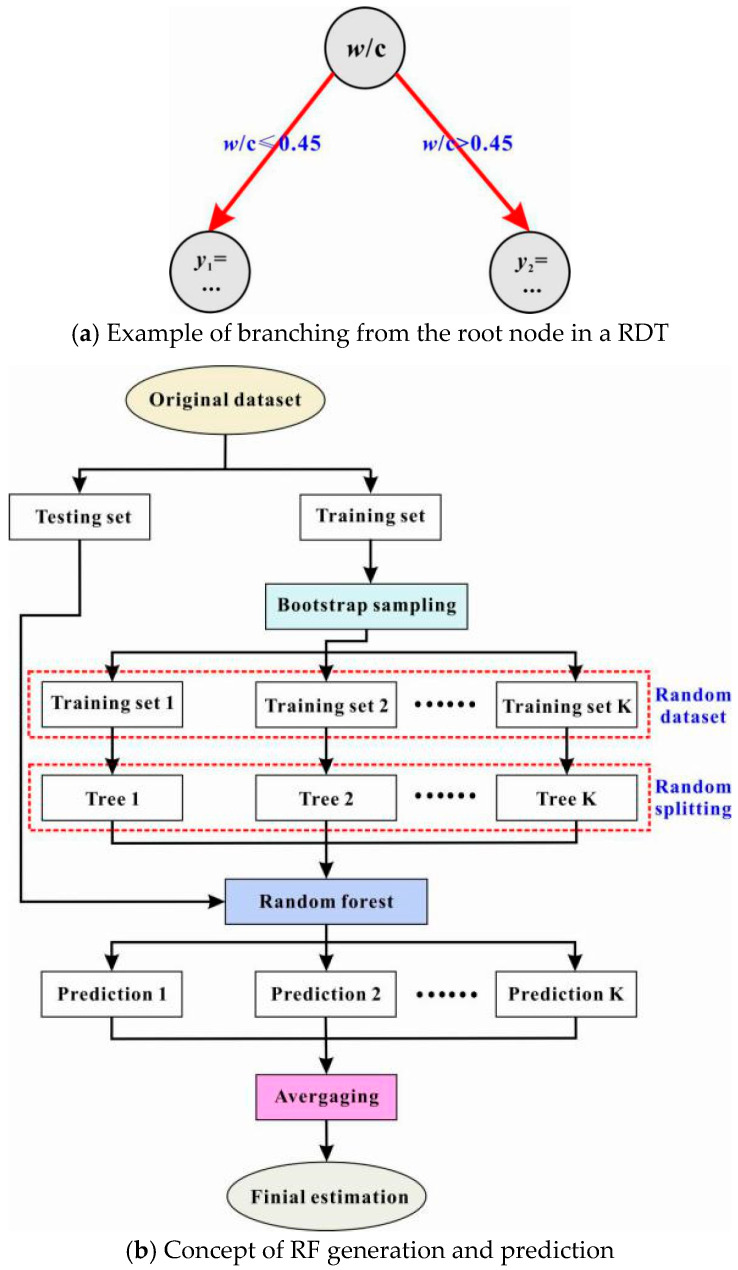
Schematic illustrations of regression decision tree (RDT) and random forest (RF).

Piece-wise regression is usually adopted to explain the mathematical features of RDT, in which the exact regression equation used to evaluate a given dataset depends on the parameter features of the dataset and the tree structure. To aid a clear description of RDT, an example tree using the water-to-cement ratio parameter of a dataset about RAC specimens to predict their compressive strength is shown in Figure 4a. In that figure, each circle represents a node in the tree. The uppermost node is known as the root node, while the two nodes below are the leaf nodes. The arrows emanating from the root node are arcs. As seen in the figure, only binary splits of a root node (or other non-leaf nodes) are allowed. The tree can grow downward in that way (as shown in Figure 4a) continuously.

In an RDT, the prediction is proceeded by starting at the root node and then flowing down to the leaf nodes. Each non-leaf node of the tree can be thought of as a question about the dataset, and each arc is a possible answer to the question being asked. Taking the simple RDT in Figure 4a as an instance, the root node examines the water-to-cement ratio of each data point. If that ratio is less than or equal to a threshold (e.g., 0.45), the procedure travels down the left branch of the tree; otherwise the right branch. Such a kind of decision (i.e., asking and answering) is repeated until arriving at a leaf node. A solution is finally obtained once all leaf nodes are reached [78].

Commonly, the RDT works by memorizing the training data. This however leads to a failure to recognize the real patterns of the dataset; that is to say, the well-known phenomenon “overfitting” occurs [79]. To tackle this problem, many decision trees can be combined to provide better predictions. This advanced concept is known as RF (Figure 4b), which averages the results of many trees to overcome the poor performance of any single tree. For this reason, RF is a representative of the state-of-the-art ensemble methods [80]. Beyond the split criterion and the maximum tree depth for a single tree, RF has several more parameters like the tree number in a forest and whether or not to utilize the bootstrapping strategy for sampling trees from the forest. More details of the principle of RF can be found in [78,80]

The present study employed the RF approach which has been built in the M5PrimeLab Matlab toolbox [81]. The following parameters were used during the calculation: the total number of trees in the forest was set as 100; the bootstrap sampling method was adopted; the maximum depth of each tree was 9; no pruning and no smoothing were applied to one single tree; the minimum observation number a node has to be considered for splitting was 5; the minimum number of training observations a leaf node may represent was set as 1; and the split threshold was set as 1 × 10^−6^.

### 4.3. Prediction Results

As stated, in the present study the ML computing procedures were both implemented in Matlab [77]. It is noted that there are no acceptable generic rules to determine the size of the training data. In this study, the database presented in Appendix A Table A1 was randomly partitioned, with a ratio of 3:1, into a training set for model building and a testing set for model assessment. 

To evaluate the predictive performances of the ANN and RF models developed in this study, the root mean squared error (RMSE), the mean absolute percentage error (MAPE) and the correlation coefficient (*R*^2^) were adopted to examine the model prediction errors. The following equations are the mathematical expressions of RMSE, MAPE and *R*^2^:

(12) $$\mathrm{RMSE}=\sqrt{\frac{\sum_{i}^{N}{\left(p_{i}-y_{i}\right)}^{2}}{N}};\;\mathrm{MAPE}=\frac{1}{N}\sum_{i}^{N}{\left(\frac{p_{i}-y_{i}}{y_{i}}\right)}^{2};\;R^{2}=1-\frac{\sum_{i}^{N}{\left(p_{i}-y_{i}\right)}^{2}}{\sum_{i}^{N}{\left(y_{i}-y^{\mathrm{avg}}\right)}^{2}}$$

where *p* and *y* respectively represent the predicted outputs and the measured outputs; *y*^avg^ is the average value of the measured outputs; and *N* is the total number of the training and testing records.

**Figure 5:**
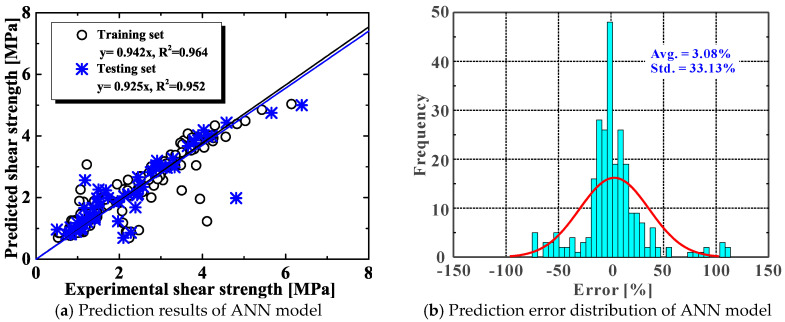

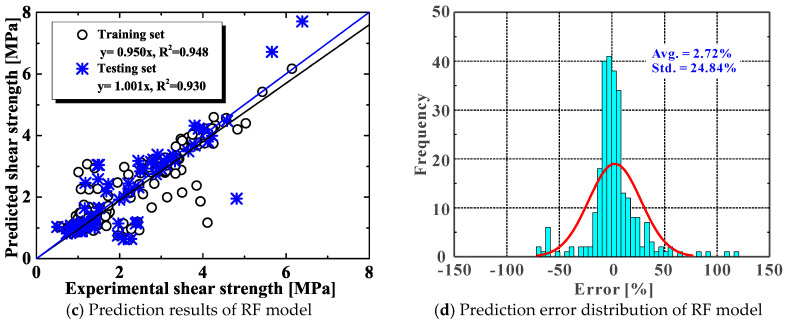
Prediction results and error distributions of the two machine learning (ML) models.

**Figure 6:**
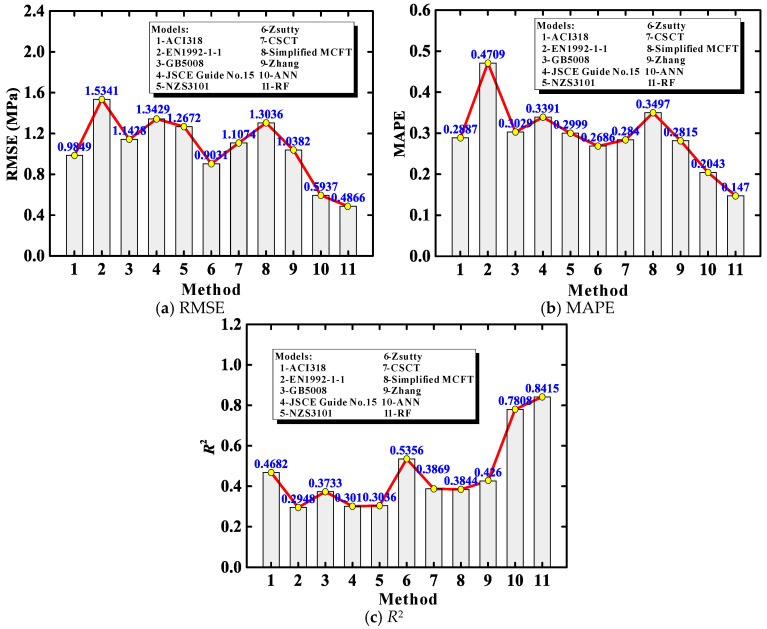
Predictive performance evaluation for different models.

In order to develop unified and versatile predictive modeling approaches, the experimental database was not split according to the presence of steel stirrups (as previously done for evaluating the existing shear design methods). Figure 5 shows the comparison of the shear strength between the ML predictions and the experimental measurements. It is noteworthy that lower RMSE and MAPE and higher *R*^2^ values indicate a better accuracy of the model prediction [48]. Figure 6 compares the results of the performance indicators for the two ML-based approaches and the previously evaluated methods. It can be seen from the above figures that, the ANN and RF models have the lowest RMSE and MAPE and the highest *R*^2^ values compared to the nine existing methods, indicating that the ML models provide better predictive results in identifying the shear capacity of RAC beams. Moreover, the RF model slightly outperforms the ANN model, due mainly to the former’s superior decision-making capacity and bootstrapping strategy.

From Figure 1 and Figure 5, it is also found that: (*i*) the average relative errors of the nine existing methods are all lower than zero (varying from –47.27% to –8.97%), suggesting various extents of conservatism. Comparatively, the average relative errors for the ML approaches are closer to zero (3.08% and 2.72%, respectively), representing higher accuracy but sacrificing a bit of conservatism; (*ii*) for the prediction variability (i.e., precision) concerned, the two ML approaches surpass all of the existing methods. Only a small portion of data is not well captured by the ML approaches.

## 5. Parametric Study with Reference to the Shear Test Database

As presented in Section 4, the developed ANN and RF models can be used to realistically simulate the shear capacity of RAC beams. In the current section, the results of a parametric study using the two models are presented to understand how exactly the shear capacity of the RAC beams is influenced by the parameters. In doing this, the compiled test database is referenced consistently for validation.

### 5.1. Effect of the Yield Strength and Amount of Shear Reinforcement

For NAC beams, the yield strength and the volumetric ratio of shear reinforcement (i.e., *f*_y_ and *ρ*_sv_, respectively) have a similar effect on the shear capacity [82]. To see if the same effect holds, four RAC beams were simulated using the ML approaches by varying the combined parameter *ρ*_sv_*f*_yv_ from 0 to 3.0 (specifically, *ρ*_sv_*f*_yv_ = 0, 0.5, 1.5 and 3.0). During the simulation, it was followed that: (*i*) the above range of *ρ*_sv_*f*_yv_ (0–3.0) for the simulation was kept to be similar to that of the test database and (*ii*) at the same time, other input parameters in the ML approaches were assigned approximately as their experimental mean values, i.e., *f*_c_ = 30 MPa, *r* = 50%, *s*_max_ = 25 mm, *a*/*d* = 2.5, *h* = 300 mm and *ρ*_s_ = 1.5%.

Group I of Table 2 shows the predicted nominal ultimate shear stress *v*_u_ using different models (Model 1–11 in the table) as *ρ*_sv_*f*_yv_ varies. When using the nine existing methods, the predicted value of *v*_u_ was just linearly proportional to the parameter *ρ*_sv_*f*_yv_, while a nonlinear dependence of *v*_u_ on *ρ*_sv_*f*_yv_ (i.e., the increasing rate of *v*_u_ gradually slows down with increasing *ρ*_sv_*f*_yv_) is observed for the two ML approaches. Obviously, the ML approaches are more reasonable and consistent with the test results.

### 5.2. Effects of the RAC Compressive Strength and the Replacement Ratio of RA

Four compressive strengths of RAC (*f*_c_ = 20 MPa, 30 MPa, 40 MPa and 50 MPa) at *r* = 50% were used to check the effect of the RAC strength on the ultimate shear resistance. The other parameters were set as: *s*_max_ = 25 mm, *a*/*d* = 2.5, *h* = 300 mm, *ρ*_s_ = 1.5% and *ρ*_sv_*f*_yv_ = 0.5. Group II of Table 2 shows the predictive results of *v*_u_ when *f*_c_ varies. As expected, all the ten methods correctly reflect the trend, that is, the shear capacity increases with increasing the concrete strength.

**Figure 7:**
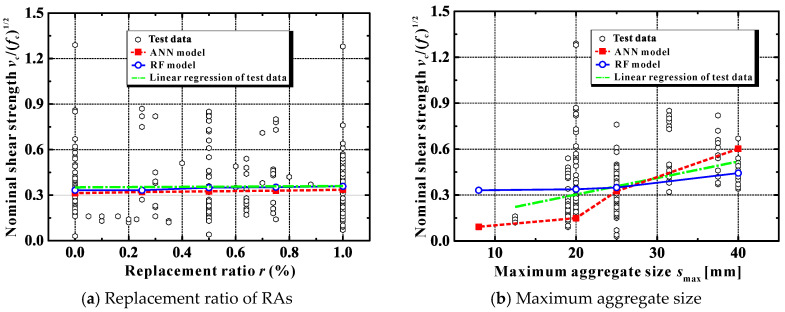

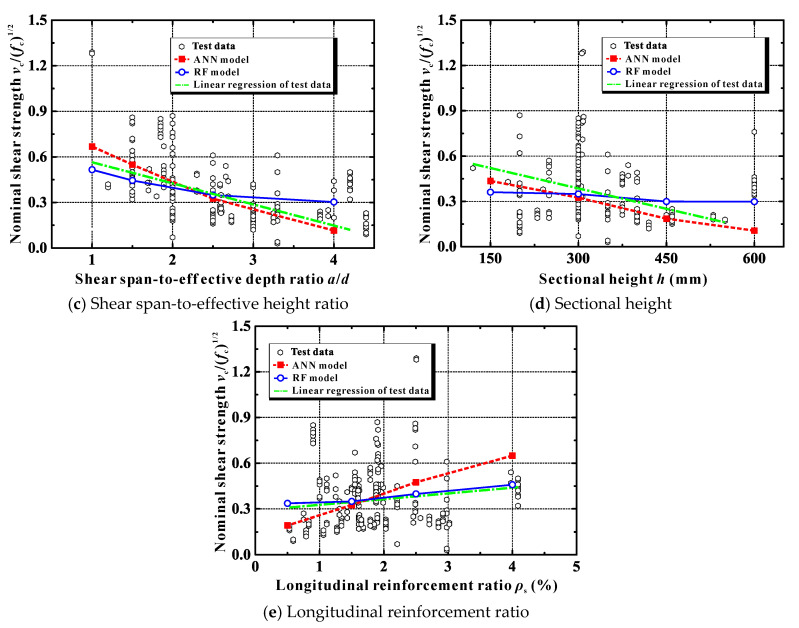
Effect of different parameters on the nominal ultimate shear stress.

As far as the replacement ratio is concerned, five additional beams were modeled using *r* = 0, 25%, 50%, 75% and 100% respectively. The remaining parameters were as follows: *f*_c_ = 30 MPa, *s*_max_ = 25 mm, *a*/*d* = 2.5, *h* = 300 mm, *ρ*_s_ = 1.5% and *ρ*_sv_*f*_yv_ = 0.5. Group III of Table 2 reports the predicted results. Figure 7a also presents the variation of the normalized parameter *v*_c_/(*f*_c_)^1/2^ as a function of *r*, where *v*_c_ is the shear strength contributed by the concrete (obtained by *v*_u_ – *ρ*_sv_*f*_yv_). The experimental datasets, their linear regression line, together with the predicted results using the ANN and RF, are all included in the figure.

It is noticed that: (*i*) the prediction results of the ML models for the shear capacity of RAC beams are generally consistent with the linear regression line of the test data; (*ii*) when the concrete contribution *v*_c_ is normalized with respect to the square root of concrete strength, the two predicted trends using the ML approaches seem almost independent of the replacement ratio of RA. The experimental scatter also indicates that there is no definite correlation between *v*_c_/(*f*_c_)^1/2^ and the replacement level. Note that when the concrete contribution is normalized, the difference of the resulting values (*v*_c_/(*f*_c_)^1/2^) between different replacement ratios tends to get limited. This has been experimentally evidenced by the investigation conducted by Ignjatovic´et al. [32], where only 5% difference was found when comparing the *v*_c_/(*f*_c_)^1/2^ values among the beams with 0%, 50% and 100% replacement ratios. When more test data are assembled (as done in this paper), it does become more difficult to draw quantitative conclusions on the relationship between *v*_c_/(*f*_c_)^1/2^ and the RA replacement level.

Still, it should be emphasized that as the scatter plot of the test data (Figure 7a) indicates, large experimental scatter exhibits for all of the replacement ratios, even when *r* is equal to zero. This condition highlights the most sophisticated and challenging part of the shear resistance prediction for concrete beams (i.e., capturing the experimental randomness). Though the ANN and RF models agree with the average trend line (the existing design methods nonetheless underestimate it), the uncertainty is still a big problem. In this sense, it does require more experimental results to reinforce the conclusion [40].

Overall, it is safe to say that, within the scope of the current test database, the ML approaches provide statistically accurate shear strength predictions, whether the replacement ratio is 0 or 100%. In addition, the above explanations justify that, in order to reduce the uncertainty and to achieve a similar safety margin between RAC and NAC beams, design-oriented treatments such as using partial factors calibrated to account for the detrimental influence of RA inclusion are reasonable and can be applied when determining the shear strength of RAC beams (as investigated in [40]).

### 5.3. Effect of the Maximum Size of RA

Previous studies have found that larger coarse aggregates improve the shear capacity of beams due to enhanced interlock action (e.g., [60,66,79]). The effect of the maximum size of RA was investigated by varying the value of *s*_max_ from 8 mm to 25 mm (see Group IV of Table 2 for detail). The other input parameters were chosen as: *f*_c_ = 30 MPa, *r* = 50%, *a*/*d* = 2.5, *h* = 300 mm, *ρ*_s_ = 1.5% and *ρ*_sv_*f*_yv_ = 0.5.

Figure 7b shows the effect of *s*_max_ on *v*_c_/(*f*_c_)^1/2^. It can be identified that, for RAC beams, the beneficial effect of the larger aggregate size can be represented by both ML approaches. Note, though, that for the existing methods, only the NZS3101-2006 [65], the simplified MCFT [66,67] and the CSCT [70,71] can account for such effect, as shown in Group IV of Table 2.

### 5.4. Effect of the Shear Span-to-Effective Height Ratio

Generally, the shear span-to-effective height ratio (*a*/*d*) is one of the most influential structural factors determining the shear capacity. The effect of *a*/*d* was assessed by varying its values as 1.0, 1.5, 2.5 and 4.0 (see Group V of Table 2 for detail). The other input parameters were set as: *f*_c_ = 30 MPa, *r* = 50%, *s*_max_ = 25 mm, *h* = 300 mm, *ρ*_s_ = 1.5% and *ρ*_sv_*f*_yv_ = 0.5.

Figure 7c shows the variation of *v*_c_/(*f*_c_)^1/2^ with *a*/*d*. As anticipated, the shear capacity of RAC beams decreased significantly with the increase of *a*/*d*. The prediction results of the ML approaches exhibit good correlations with the trend of the test data.

From the results of Group V in Table 2, it can be found that the JSCE Guideline No.15 [64] and the NZS3101-2006 [65] yield an identical shear capacity for all of the evaluated *a*/*d* values. Also, the GB50010-2010 [63] gives the same result when *a*/*d* is equal to 1.0 and 1.5. This manifests the inadequacy of these design standards in terms of considering the effect of the shear span ratio.

### 5.5. Effect of the Beam Height

The size effect of beam shear capacity is of significant concern [59,60], which is closely related to the beam section height. Four cases of beam height, i.e., *h* = 150 mm, 300 mm, 450 mm and 600 mm, were considered in the parametric study. The other parameters were: *f*_c_ = 30 MPa, *r* = 50%, *s*_max_ = 25 mm, *a*/*d* = 2.5, *ρ*_s_ = 1.5% and *ρ*_sv_*f*_yv_ = 0.5.

Figure 7d shows how the parameter *h* affects the normalized parameter *v*_c_/(*f*_c_)^1/2^. Evidently, the size effect that the shear capacity decreases with increasing the beam height can be well reproduced by the two ML approaches. Moreover, by inspecting the results of Group VI in Table 2, it can be found that the three design codes of ACI318-2014 [61], GB50010-2010 [63] and NZS3101-2006 [65] cannot explicitly capture the size effect on the ultimate shear strength.

### 5.6. Effect of the Longitudinal Reinforcement Ratio

As indicated by the GRA, the longitudinal reinforcement ratio has a great impact on the beams’ shear resistance. Four RAC beams with different longitudinal reinforcement ratio, i.e., *ρ*_s_ = 0.5%, 1.5%, 2.5% and 4.0%, were modeled. The other parameters were kept constant: *f*_c_ = 30 MPa, *r* = 50%, *s*_max_ = 25 mm, *a*/*d* = 2.5, *h* = 300 mm and *ρ*_sv_*f*_yv_ = 0.5.

Figure 7f shows the effect of *ρ*_s_ on *v*_c_/(*f*_c_)^1/2^. The trend is within the expectation that an increased longitudinal reinforcement ratio leads to an improved shear capacity of RAC beams, since the dowel effect becomes more significant.

As to the prediction capacity of the existing methods, the results of Group VII in Table 2 clearly show that the GB50010-2010 [63] and NZS3101-2006 [65] fall short in representing the role of the longitudinal reinforcement.

## 6. Conclusions and Remarks

This study intends to quantify the shear capacity of RAC beams by means of two typical Machine Learning approaches (the artificial neural network (ANN) and the random forest (RF)). The model training and validation are based on 264 RAC beam shear tests collated from the open literature. Nine existing shear design methods are also appraised. The following conclusions can be drawn base on the presented investigation results:

(1) The existing design methods show conservative, inaccurate shear strength estimations for RAC beams with large scatter, especially for RAC beams without stirrups. These methods generally cannot take full account of all the influential parameters investigated in this study. The simplified MCFT and CSCT models are relatively comprehensive, but the effect of the replacement ratio of RA is not (of course) considered in both;
(2) The parametric importance is ranked by the GRA, which shows that the longitudinal reinforcement ratio, the maximum size of RA and the RAC compressive strength are the most relevant parameters that affect the shear resistance of RAC beams; the beam section height, the shear span ratio, and the yield strength and volumetric ratio of shear reinforcement have the secondary importance; the significance of the replacement ratio of RA comes last of all, but it is still considerable since its related Grey relational factor is over 70%;
(3) The developed ANN and RF models provide better predictive outputs than the nine existing methods, with the R^2^ above 0.930. These two models are easy-to-implement and bypass complicated theoretical derivations and numerical implementations, thus can serve as alternative predictors for designing RAC components;
(4) A parametric study is conducted using the two developed ML approaches. The results show that the ML approaches can correctly reflect the effects of the main parameters on the shear capacity of RAC beams. This is also confirmed by the experimental trends. In particular, once the shear contribution from RAC (i.e., *v*_c_) is normalized with respect to the square root of its strength, the resulting expression (*v*_c_/(*f*_c_)^1/2^) of the shear capacity of RAC beams appears to, at least in a statistical sense, be insignificantly affected by the replacement ratio of RA. This implies that the shear design of RAC beams can be processed almost the same as that of NAC beams, as long as the compressive strength of RAC is specified and guaranteed. Note that this conclusion is valid only within the scope of the documented experimental database. Furthermore, for safety purposes and cautious application, design-oriented treatments such as using partial factors adjusted to account for the uncertainty of RA inclusion are practical when determining the shear strength of RAC beams (as studied in [40]).

Despite the above-achieved results, more works need to be done in the future, which may include the following:

- The present ML-based study relies heavily upon an overall limited number of RAC beam shear tests. As emphasized by the previous study [40], test data of RAC beams with stirrups is still scant. Also, the large scatter presented in the plots of this study (such as Figure 7a) exposes the lack of new experimental evidence. Thus more relevant tests are required to consolidate the findings, including the beams incorporating more heterogeneous CDW aggregates and the beams of full-scale size; those tests not only can come in handy, but also are the basis of more robust, dynamically updated data-driven approaches;
- In the present study, only two ML models are investigated. In the future, more advanced ML models or other ensemble methods (besides the Random Forest) [80] should be considered. The approaches that can give explicit design equations (e.g., the gene expression programming method [83]) also deserve to be paid more attention. This effort can partially overcome the “black box” disadvantage of the ML models. Furthermore, the artificial intelligent agents are usually problem-dependent. It thus requires a priori experience and extensive comparative analyses. This also direct future studies. Finally, it is noteworthy that the ML models attempt to optimize their predictive capabilities so as to minimize over-predictions. This shows the need for fine-tuning these models to achieve a rational balance between accuracy and conservatism;
- The ML-based models, howsoever accurate or “advanced”, are not intended to discredit conventional mechanics-based methods. In fact, the former approaches are only alternative tools at the present development stage. They provide an opportunity in this “big data” time to solve the structural problems that the conventional methods have difficulties. Ideally, the two kinds of approaches are expected to complement and support each other. For example, empirical coefficients in the conventional methods can be refined by the ML approaches. Anyway, it is still valuable and intriguing that more contributors can delve into the theoretical studies trying to reveal the hidden mechanisms of RAC beams in shear; this is probably the only way that would shed direct light on this complex problem.

Machine Learning (or more broadly, Artificial Intelligence) is a big tech buzzword in today’s industry; it is also a big challenge for policymakers, developers and users. Carefulness is surely required when using those techniques, particularly for the case where new scenarios are out of the scope of the training data. Due to the limitation of space, more details of our work cannot be presented. Readers with interest are encouraged to contact the authors for more information or discussion.

## Figures and Tables

**Table 1:** Statistics of parameters in the database.

Parameter	*f*_c_ [MPa]	*r*	*s*_max_ [mm]	*a*/*d*	*h* [mm]	*ρ*_s_	*ρ*_sv_*f*_yv_ [MPa]	*v*_u_ [MPa]
Maximum	49.8	1.00	40.0	5.00	680.0	0.0409	3.3743	6.39
Mean	32.7	0.57	24.5	2.71	349.0	0.0181	0.2355	2.27
Minimum	16.8	0.00	12.5	1.00	120.0	0.0053	0.0000	0.69
Standard deviation	6.9	0.40	6.8	0.93	114.5	0.0079	0.5095	1.25
Standard error	0.5	0.03	0.5	0.06	7.9	0.0005	0.0352	0.09
Median	32.6	0.50	25.0	2.55	300.0	0.0165	0.0000	1.36
Mode	29.0	1.00	25.0	3.00	300.0	0.0161	0.0000	2.43
Kurtosis	−0.6	−1.49	0.4	−0.28	0.7	0.8177	7.8658	−0.23
Skewness	0.3	−0.25	0.9	0.62	0.9	0.8972	2.5641	0.71

**Table 2:** Predicted nominal ultimate shear stress *v*_u_ using different models.

Group	Input Parameters	Model No.										
No.	[*f*_c_, *r*, *s*_max_, *a*/*d*, *h*, *ρ*_s_, *ρ*_sv_*f*_yv_]	1	2	3	4	5	6	7	8	9	10	11
I	[30, 0.50, 25, 2.5, 300, 1.5%, 0.0]	1.23	1.02	0.72	1.18	0.99	1.18	1.01	1.35	1.23	1.43	1.80
	[30, 0.50, 25, 2.5, 300, 1.5%, 0.5]	1.73	1.52	1.22	1.68	1.49	1.68	1.51	1.85	1.73	2.28	2.41
	[30, 0.50, 25, 2.5, 300, 1.5%, 1.5]	2.73	2.52	2.22	2.68	2.49	2.68	2.51	2.85	2.73	3.20	2.80
	[30, 0.50, 25, 2.5, 300, 1.5%, 3.0]	4.23	4.02	3.72	4.18	3.99	4.18	4.01	4.35	4.23	3.76	2.90
II	[20, 0.50, 25, 2.5, 300, 1.5%, 0.5]	1.57	1.35	1.09	1.29	1.37	1.46	1.48	1.80	1.50	1.18	2.33
	[30, 0.50, 25, 2.5, 300, 1.5%, 0.5]	1.73	1.52	1.22	1.68	1.49	1.68	1.51	1.85	1.73	2.28	2.41
	[40, 0.50, 25, 2.5, 300, 1.5%, 0.5]	1.85	1.66	1.31	2.01	1.59	1.86	1.53	1.90	1.92	3.18	2.64
	[50, 0.50, 25, 2.5, 300, 1.5%, 0.5]	1.96	1.78	1.39	2.31	1.68	2.02	1.55	1.93	2.09	3.28	2.79
III	[30, 0.00, 25, 2.5, 300, 1.5%, 0.5]	1.73	1.52	1.22	1.68	1.49	1.68	1.51	1.82	1.73	2.22	2.32
	[30, 0.25, 25, 2.5, 300, 1.5%, 0.5]	1.73	1.52	1.22	1.68	1.49	1.68	1.51	1.85	1.73	2.25	2.32
	[30, 0.50, 25, 2.5, 300, 1.5%, 0.5]	1.73	1.52	1.22	1.68	1.49	1.68	1.51	1.85	1.73	2.28	2.41
	[30, 0.75, 25, 2.5, 300, 1.5%, 0.5]	1.73	1.52	1.22	1.68	1.49	1.68	1.51	1.85	1.73	2.30	2.43
	[30, 1.00, 25, 2.5, 300, 1.5%, 0.5]	1.73	1.52	1.22	1.68	1.49	1.68	1.51	1.85	1.73	2.34	2.46
IV	[30, 0.50, 8, 2.5, 300, 1.5%, 0.5]	1.73	1.52	1.22	1.68	1.49	1.40	1.18	1.35	1.73	1.01	2.31
	[30, 0.50, 20, 2.5, 300, 1.5%, 0.5]	1.73	1.52	1.22	1.68	1.49	1.60	1.44	1.74	1.73	1.32	2.35
	[30, 0.50, 25, 2.5, 300, 1.5%, 0.5]	1.73	1.52	1.22	1.68	1.49	1.68	1.51	1.85	1.73	2.28	2.41
	[30, 0.50, 40, 2.5, 300, 1.5%, 0.5]	1.73	1.52	1.22	1.68	1.49	1.92	1.68	2.13	1.73	3.80	2.93
V	[30, 0.50, 25, 1.0, 300, 1.5%, 0.5]	4.67	1.67	1.22	2.15	1.49	1.68	1.71	2.06	2.90	4.16	3.33
	[30, 0.50, 25, 1.5, 300, 1.5%, 0.5]	2.93	2.49	1.22	2.15	1.49	1.68	1.63	1.98	2.29	3.51	2.94
	[30, 0.50, 25, 2.5, 300, 1.5%, 0.5]	1.73	1.52	1.22	1.68	1.49	1.68	1.51	1.85	1.73	2.28	2.41
	[30, 0.50, 25, 4.0, 300, 1.5%, 0.5]	1.55	1.48	1.22	1.53	1.49	1.68	1.39	1.72	1.57	1.13	2.16
VI	[30, 0.50, 25, 2.5, 150, 1.5%, 0.5]	1.73	1.52	1.39	1.68	1.57	1.68	1.62	2.00	2.36	2.88	2.48
	[30, 0.50, 25, 2.5, 300, 1.5%, 0.5]	1.73	1.52	1.22	1.68	1.49	1.68	1.51	1.85	1.73	2.28	2.41
	[30, 0.50, 25, 2.5, 450, 1.5%, 0.5]	1.73	1.52	1.15	1.68	1.39	1.67	1.46	1.75	1.53	1.52	2.14
	[30, 0.50, 25, 2.5, 600, 1.5%, 0.5]	1.73	1.52	1.11	1.68	1.32	1.58	1.41	1.67	1.42	1.08	2.13
VII	[30, 0.50, 25, 2.5, 300, 0.5%, 0.5]	1.35	1.45	1.00	1.68	1.19	1.21	1.18	1.49	1.50	1.56	2.34
	[30, 0.50, 25, 2.5, 300, 1.5%, 0.5]	1.73	1.52	1.22	1.68	1.49	1.68	1.51	1.85	1.73	2.28	2.41
	[30, 0.50, 25, 2.5, 300, 2.5%, 0.5]	1.96	1.58	1.35	1.68	1.68	1.68	1.68	2.01	1.96	3.10	2.68
	[30, 0.50, 25, 2.5, 300, 4.0%, 0.5]	2.20	1.69	1.50	1.68	1.80	1.68	1.84	2.13	2.30	4.06	3.01

Note: (1) During the above calculations, the sectional width and concrete cover of each beam were supposed to be 150 mm and 25 mm, respectively; (2) Models 1~9 correspond, respectively, to the formulae by Zsutty [57,58], ACI318-2014 [61], EN1992-1-1:2004 [62], GB50010-2010 [63], JSCE Guideline No.15 [64], NZS3101-2006 [65], Simplified MCFT [66,67], CSCT [70,71] and Zhang [72]. Models 10~11 are the developed ANN and RF models; (3) Red numbers with a light yellow background indicate that the corresponding model contradicts the experimental trend.

## Appendix A

**Table A1:** Database of RAC beam specimens.

Source	*b* [mm]	*h* [mm]	*d* [mm]	*a*/*d*	*r*	*w*/*c*	*s*_max_ [mm]	*f*_c_ [MPa]	*ρ*_s_ [%]	*f*_y_ [MPa]	*ρ*_sv_ [%]	*f*_yv_ [MPa]	*V*_u_ [kN]
Han et al. [18]	170	300	270	1.50	1.00	0.40	25.0	39.6	1.11	430	0	–	144.1
	170	300	270	2.00	1.00	0.40	25.0	30.6	1.11	430	0	–	117.5
	170	300	270	3.00	1.00	0.40	25.0	31.2	1.11	430	0	–	55.1
	170	300	270	4.00	1.00	0.40	25.0	31.9	1.11	430	0	–	50.9
	170	300	270	2.00	1.00	0.40	25.0	32.6	1.11	430	0	–	113.4
	170	300	270	2.00	0	0.40	25.0	37.4	1.11	430	0	–	118.0
	170	300	270	2.00	1.00	0.40	25.0	41.9	2.21	430	0.09	410	150.1
	170	300	270	2.00	1.00	0.40	25.0	41.1	2.21	430	0.24	410	153.3
	170	300	270	2.00	1.00	0.40	25.0	31.6	2.21	430	0.51	410	174.4
	170	300	270	2.00	1.00	0.40	25.0	41.1	2.21	430	0.82	410	174.4
	170	300	270	2.00	1.00	0.40	25.0	37.4	2.21	430	0.24	410	141.8
	170	300	270	2.00	0	0.40	25.0	49.8	2.21	430	0.24	410	154.2
Etxeberria et al. [19]	200	350	300	3.3	0	0.55	25	41.9	2.92	500	0	–	100.5
	200	350	300	3.3	0.25	0.55	25	42.4	2.92	500	0	–	104.0
	200	350	300	3.3	0.50	0.52	25	41.3	2.92	500	0	–	89.0
	200	350	300	3.3	1.00	0.55	25	41.9	2.92	500	0	–	84.0
González & Martínez [20]	200	350	303	3.30	0	0.55	25.0	40.2	2.98	571	0	–	190.3
	200	350	303	3.30	0.50	0.55	25.0	39.7	2.98	571	0	–	233.6
	200	350	303	3.30	0	0.55	25.0	39.2	2.98	571	0.12	571	150.8
	200	350	303	3.30	0.50	0.55	25.0	39.2	2.98	571	0.12	571	177.0
	200	350	303	3.30	0	0.55	25.0	39.1	2.98	571	0.17	571	128.0
	200	350	303	3.30	0.50	0.55	25.0	41.5	2.98	571	0.17	571	164.3
	200	350	303	3.30	0	0.55	25.0	37.7	2.98	571	0.22	571	88.9
	200	350	303	3.30	0.50	0.55	25.0	40.5	2.98	571	0.22	571	90.6
Fathifazl et al. [21,22]	200	375	309	2.59	0.64	0.45	19.0	36.9	1.43	407	0	–	104.0
	200	375	306	2.61	0.64	0.45	19.0	36.9	2.57	418	0.25	530	172.0
	200	385	301	2.66	0.64	0.45	19.0	36.9	3.98	442	0.5	473	341.0
	200	385	301	2.66	0.74	0.45	19.0	43.5	3.98	442	0.5	473	327.0
	200	375	309	2.59	0	0.45	19.0	38.0	1.43	407	0	–	93.0
	200	375	309	2.59	0	0.45	19.0	35.9	1.43	407	0	–	150.0
Choi et al. [23]	200	400	360	1.50	0	0.50	25.0	24.7	1.61	456	0	–	176.4
	200	400	360	2.50	0	0.50	25.0	24.7	1.61	456	0	–	90.7
	200	400	360	3.25	0	0.50	25.0	24.7	1.61	456	0	–	71.1
	200	400	360	1.50	0.30	0.50	25.0	24.6	1.61	456	0	–	161.7
	200	400	360	2.50	0.30	0.50	25.0	24.6	1.61	456	0	–	81.3
	200	400	360	3.25	0.30	0.50	25.0	24.6	1.61	456	0	–	80.9
	200	400	360	1.50	0.50	0.50	25.0	24.2	1.61	456	0	–	152.9
	200	400	360	2.50	0.50	0.50	25.0	24.2	1.61	456	0	–	87.9
	200	400	360	3.25	0.50	0.50	25.0	24.2	1.61	456	0	–	71.5
	200	400	360	1.50	1.00	0.50	25.0	22.6	1.61	456	0	–	107.8
	200	400	360	2.50	1.00	0.50	25.0	22.6	1.61	456	0	–	84.8
	200	400	360	3.25	1.00	0.50	25.0	22.6	1.61	456	0	–	57.8
	200	400	360	2.50	0	0.50	25.0	24.7	0.53	522	0	–	66.2
	200	400	360	2.50	0	0.50	25.0	24.7	0.83	486	0	–	72.0
	200	400	360	2.50	0	0.50	25.0	24.7	1.61	456	0	–	90.7
	200	400	360	2.50	0.30	0.50	25.0	24.6	0.53	522	0	–	56.7
	200	400	360	2.50	0.30	0.50	25.0	24.6	0.83	486	0	–	78.4
	200	400	360	2.50	0.30	0.50	25.0	24.6	1.61	456	0	–	81.3
	200	400	360	2.50	0.50	0.50	25.0	24.2	0.53	522	0	–	57.8
	200	400	360	2.50	0.50	0.50	25.0	24.2	0.83	486	0	–	67.1
	200	400	360	2.50	0.50	0.50	25.0	24.2	1.61	456	0	–	87.7
	200	400	360	2.50	1.00	0.50	25.0	22.6	0.53	522	0	–	59.8
	200	400	360	2.50	1.00	0.50	25.0	22.6	0.83	486	0	–	70.1
	200	400	360	2.50	1.00	0.50	25.0	22.6	1.61	456	0	–	84.8
Fathifazl et al. [24]	200	375	300	1.50	0.64	0.45	19.0	41.6	1.00	418	0	–	186.7
	200	375	300	2.00	0.64	0.45	19.0	41.6	1.50	418	0	–	169.5
	200	375	309	2.59	0.64	0.45	19.0	41.6	1.62	407	0	–	103.9
	200	375	309	2.59	0	0.45	19.0	38.0	1.62	407	0	–	92.8
	200	375	305	3.93	0.64	0.45	19.0	41.6	2.46	418	0	–	83.2
	200	375	300	1.50	0.74	0.45	19.0	49.1	1.00	418	0	–	>195.3
	200	375	300	2.00	0.74	0.45	19.0	49.1	1.50	418	0	–	179.0
	200	375	309	2.59	0	0.45	19.0	34.1	1.62	407	0	–	150.0
	200	375	305	3.93	0.74	0.45	19.0	49.1	2.46	418	0	–	105.6
	200	250	201	2.69	0.64	0.45	19.0	41.6	1.99	416	0	–	89.3
	200	375	309	2.59	0.64	0.45	19.0	41.6	1.62	407	0	–	103.9
	200	375	309	2.59	0	0.45	19.0	38.0	1.62	407	0	–	92.8
	200	450	381	2.73	0.64	0.45	19.0	41.6	1.83	425	0	–	99.5
	200	550	476	2.73	0.64	0.45	19.0	41.6	1.68	426	0	–	104.6
	200	250	201	2.69	0.74	0.45	19.0	49.1	1.99	416	0	–	>122.6
	200	375	309	2.59	0	0.45	19.0	34.1	1.62	407	0	–	>150.0
	200	450	381	2.73	0.74	0.45	19.0	49.1	1.83	425	0	–	111.7
	200	550	476	2.73	0.74	0.45	19.0	49.1	1.68	426	0	–	119.6
Arezoumandi et al. [25,26]	300	460	407	3.00	0	0.40	25.0	37.2	1.27	414	0	–	121.0
	300	460	407	3.00	0	0.40	25.0	34.1	1.27	414	0	–	129.9
	300	460	407	3.00	0	0.40	25.0	37.2	2.03	414	0	–	143.2
	300	460	407	3.00	0	0.40	25.0	34.1	2.03	414	0	–	166.8
	300	460	407	3.00	0	0.40	25.0	37.2	2.71	414	0	–	173.5
	300	460	407	3.00	0	0.40	25.0	34.1	2.71	414	0	–	170.8
	300	460	407	3.00	0.50	0.40	25.0	32.0	1.27	414	0	–	117.4
	300	460	407	3.00	0.50	0.40	25.0	35.5	1.27	414	0	–	111.6
	300	460	407	3.00	0.50	0.40	25.0	32.0	2.03	414	0	–	151.2
	300	460	407	3.00	0.50	0.40	25.0	35.5	2.03	414	0	–	148.6
	300	460	407	3.00	0.50	0.40	25.0	32.0	2.71	414	0	–	171.7
	300	460	407	3.00	0.50	0.40	25.0	35.5	2.71	414	0	–	168.6
	300	460	407	3.00	1.00	0.40	25.0	30.0	1.27	414	0	–	114.8
	300	460	407	3.00	1.00	0.40	25.0	34.1	1.27	414	0	–	113.0
	300	460	407	3.00	1.00	0.40	25.0	30.0	2.03	414	0	–	143.2
	300	460	407	3.00	1.00	0.40	25.0	34.1	2.03	414	0	–	124.1
	300	460	407	3.00	1.00	0.40	25.0	30.0	2.71	414	0	–	131.2
	300	460	407	3.00	1.00	0.40	25.0	34.1	2.71	414	0	–	140.1
Knaack & Kurama [27]	150	230	200	3.83	0	0.44	19.0	31.2	1.34	572	0	–	31.1
	150	230	200	3.83	0	0.44	19.0	31.2	1.34	572	0	–	36.9
	150	230	200	3.83	0	0.44	19.0	46.4	1.34	572	0	–	40.4
	150	230	200	3.83	0	0.44	19.0	46.4	1.34	572	0	–	42.3
	150	230	200	3.83	0.50	0.44	19.0	41.8	1.34	572	0	–	44.0
	150	230	200	3.83	0.50	0.44	19.0	41.8	1.34	572	0	–	39.1
	150	230	200	3.83	0.50	0.44	19.0	37.4	1.34	572	0	–	43.7
	150	230	200	3.83	0.50	0.44	19.0	37.4	1.34	572	0	–	41.2
	150	230	200	3.83	1.00	0.44	19.0	39.1	1.34	572	0	–	36.4
	150	230	200	3.83	1.00	0.44	19.0	39.1	1.34	572	0	–	38.0
	150	230	200	3.83	1.00	0.44	19.0	39.2	1.34	572	0	–	39.9
	150	230	200	3.83	1.00	0.44	19.0	39.2	1.34	572	0	–	36.1
Katkhuda & Shatarat [29]	206	300	260	2.00	0	0.50	20.0	28.3	1.90	457	0	–	69.3
	206	300	260	2.00	0.50	0.50	20.0	25.2	1.90	457	0	–	59.0
	206	300	260	2.00	1.00	0.50	20.0	23.2	1.90	457	0	–	55.0
	206	300	260	2.00	0.50	0.50	20.0	28.1	1.90	457	0	–	73.0
	206	300	260	2.00	1.00	0.50	20.0	26.6	1.90	457	0	–	66.8
	206	300	267	2.00	0	0.50	20.0	28.3	1.85	457	0	–	52.5
	206	300	267	2.00	0.50	0.50	20.0	25.2	1.85	457	0	–	49.1
	206	300	267	2.00	1.00	0.50	20.0	23.2	1.85	457	0	–	46.5
	206	300	267	2.00	0.50	0.50	20.0	28.1	1.85	457	0	–	55.1
	206	300	267	2.00	1.00	0.50	20.0	26.6	1.85	457	0	–	55.6
Choi and Yun [30]	400	600	525	2.0	1.00	0.44	25.0 *	29.0	1.88	433	0	–	862.7
	400	600	525	2.5	1.00	0.44	25.0 *	29.0	1.88	433	0	–	518.7
	400	600	525	3.0	1.00	0.44	25.0 *	29.0	1.88	433	0	–	454.2
	400	600	525	3.0	1.00	0.44	25.0 *	29.0	1.88	433	0	–	478.0
	400	600	525	4.0	1.00	0.44	25.0 *	29.0	1.88	433	0	–	501.8
	400	600	525	4.0	1.00	0.44	25.0 *	29.0	1.88	433	0	–	434.0
	400	600	525	5.0	0	0.44	25.0 *	29.0	1.88	433	0	–	460.3
	400	600	525	5.0	0	0.44	25.0 **	37.0	1.88	433	0	–	440.5
	400	600	525	5.0	0.30	0.44	25.0 *	34.0	1.88	433	0	–	470.9
	400	600	525	5.0	0.30	0.44	25.0 *	34.0	1.88	433	0	–	478.4
	400	600	525	5.0	0.60	0.44	25.0 *	32.0	1.88	433	0	–	411.8
	400	600	525	5.0	0.60	0.44	25.0 *	32.0	1.88	433	0	–	412.2
	400	600	525	5.0	1.00	0.44	25.0 *	29.0	1.88	433	0	–	413.0
	400	600	525	5.0	1.00	0.44	25.0 *	29.0	1.88	433	0	–	438.6
Rahal & Alrefaei [31]	150	420	388	3.00	0	0.50	12.5	35.6	0.79	534	0	–	54.5
	150	420	388	3.00	1.00	0.54	12.5	32.2	0.79	534	0	–	42.5
	150	420	388	3.00	0.10	0.50	12.5	34.2	0.79	534	0	–	44.5
	150	420	388	3.00	0.20	0.51	12.5	33.4	0.79	534	0	–	40.1
	150	420	388	3.00	0.20	0.51	12.5	34.7	0.79	534	0	–	48.9
	150	420	388	3.00	0.35	0.52	12.5	33.7	0.79	534	0	–	45.1
	150	420	388	3.00	0.50	0.53	12.5	36.2	0.79	534	0	–	47.0
	150	420	388	3.00	0.75	0.53	12.5	35.7	0.79	534	0	–	47.4
	150	420	388	3.00	0.05	0.50	12.5	37.6	0.79	534	0	–	56.0
	150	420	388	3.00	0.10	0.53	12.5	33.3	0.79	534	0	–	52.5
	150	420	388	3.00	0.16	0.53	12.5	33.2	0.79	534	0	–	54.2
	150	420	388	3.00	0.23	0.53	12.5	32.8	0.79	534	0	–	47.3
	150	420	388	3.00	0.35	0.51	12.5	35.3	0.79	534	0	–	42.5
Ignjatović et al. [32]	200	300	235	4.20	0	0.60	31.5	33.3	4.09	560	0	–	106.3
	200	300	235	4.20	0.50	0.60	31.5	37.0	4.09	560	0	–	91.8
	200	300	235	4.20	1.00	0.58	31.5	33.9	4.09	560	0	–	104.8
	200	300	235	4.20	0	0.60	31.5	33.3	4.09	560	0.14	300	140.7
	200	300	235	4.20	0.50	0.60	31.5	37.0	4.09	560	0.14	300	142.0
	200	300	235	4.20	1.00	0.58	31.5	33.9	4.09	560	0.14	300	135.0
	200	300	235	4.20	0	0.60	31.5	33.3	4.09	560	0.19	300	160.0
	200	300	235	4.20	0.50	0.60	31.5	37.0	4.09	560	0.19	300	156.9
	200	300	235	4.20	1.00	0.58	31.5	33.9	4.09	560	0.19	300	163.4
Pradhan et al. [33]	200	300	269	2.60	0	0.45	20.0	42.8	0.75	591	0	–	94.1
	200	300	269	2.60	0	0.45	20.0	42.8	0.75	591	0	–	94.9
	200	300	269	2.60	0	0.45	20.0	42.8	1.31	519	0	–	107.2
	200	300	269	2.60	1.00	0.45	20.0	42.8	0.75	591	0	–	81.1
	200	300	269	2.60	1.00	0.45	20.0	42.8	0.75	591	0	–	81.3
	200	300	269	2.60	1.00	0.45	20.0	42.8	1.31	519	0	–	92.3
	200	300	269	2.60	1.00	0.45	20.0	42.8	1.31	519	0.20	352	161.9
	200	300	269	2.60	1.00	0.45	20.0	42.8	1.31	519	0.20	352	162.1
Wardeh et al. [34]	200	250	225	1.50	0	0.49	20.0	37.3	1.79	600	0	–	>150.0
	200	250	225	3.00	0	0.49	20.0	37.3	1.79	600	0	–	63.6
	200	250	225	3.00	0	0.49	20.0	37.3	1.79	600	0	–	60.6
	200	250	225	3.00	0	0.49	20.0	37.3	1.79	600	0	–	60.0
	200	250	225	1.50	1.00	0.41	20.0	34.5	1.79	600	0	–	130.0
	200	250	225	1.50	1.00	0.41	20.0	34.5	1.79	600	0	–	150.3
	200	250	225	1.50	1.00	0.41	20.0	34.5	1.79	600	0	–	140.4
	200	250	225	3.00	1.00	0.41	20.0	34.5	1.79	600	0	–	50.2
	200	250	225	3.00	1.00	0.41	20.0	34.5	1.79	600	0	–	49.0
Sato et al. [35]	150	200	160	4.4	1.00	0.6	19.0	46.5	1.06	331	0	–	21.0
	150	200	160	4.4	1.00	0.45	19.0	32.9	1.06	331	0	–	21.7
	150	200	160	4.4	1.00	0.60	19.0	46.6	1.06	331	0	–	21.4
	150	200	160	4.4	1.00	0.60	19.0	30.4	0.59	331	0	–	12.1
	150	200	160	4.4	1.00	0.60	19.0	28.4	0.59	331	0	–	12.6
	150	200	160	4.4	1.00	0.60	19.0	34.5	0.59	331	0	–	13.2
	150	200	160	4.4	1.00	0.60	19.0	31.8	0.59	331	0	–	13.5
	150	200	160	4.4	1.00	0.60	19.0	30.4	1.06	331	0	–	19.7
	150	200	160	4.4	1.00	0.60	19.0	28.4	1.06	331	0	–	20.0
	150	200	160	4.4	1.00	0.60	19.0	34.5	1.06	331	0	–	20.0
	150	200	160	4.4	1.00	0.60	19.0	31.8	1.06	331	0	–	21.4
	150	200	160	4.4	1.00	0.60	19.0	30.4	1.65	342	0	–	27.3
	150	200	160	4.4	1.00	0.60	19.0	28.4	1.65	342	0	–	27.7
	150	200	160	4.4	1.00	0.60	19.0	34.5	1.65	342	0	–	28.3
	150	200	160	4.4	1.00	0.60	19.0	31.8	1.65	342	0	–	31.1
Kim et al. [36]	200	350	300	2.5	0	0.46	25.0	31.8	2.85	651	0	–	75.5
	200	530	450	2.5	0	0.46	25.0	31.8	2.85	610	0	–	106.9
	200	680	600	2.5	0	0.46	25.0	31.8	2.85	651	0	–	125.9
	300	530	450	2.5	0	0.46	25.0	31.8	3.02	600	0	–	156.7
	400	680	600	2.5	0	0.46	25.0	31.8	2.85	651	0	–	256.4
	200	350	300	2.5	0.50	0.46	25.0	32.6	2.85	651	0	–	60.6
	200	530	450	2.5	0.50	0.46	25.0	32.6	2.85	610	0	–	108.9
	200	680	600	2.5	0.50	0.46	25.0	32.6	2.85	651	0	–	126.1
	300	530	450	2.5	0.50	0.46	25.0	32.6	3.02	600	0	–	154.2
	400	680	600	2.5	0.50	0.46	25.0	32.6	2.85	651	0	–	261.5
	200	350	300	2.5	1.00	0.45	25.0	34.9	2.85	651	0	–	72.9
	200	530	450	2.5	1.00	0.45	25.0	34.9	2.85	610	0	–	96.4
	200	680	600	2.5	1.00	0.45	25.0	34.9	2.85	651	0	–	125.1
	300	530	450	2.5	1.00	0.45	25.0	34.9	3.02	600	0	–	159.8
	400	680	600	2.5	1.00	0.45	25.0	34.9	2.85	651	0	–	256.6
Al-Zahraa et al. [37]	100	200	180	2.0	0	0.45	20.0	31.7	1.90	560	0	–	63.0
	100	200	180	2.0	0.25	0.45	20.0	30.4	1.90	560	0	–	86.5
	100	200	180	2.0	0.50	0.45	20.0	29.6	1.90	560	0	–	71.0
Ikegawa et al. [38]	200	300	260	2.3	0	0.46	25.0 *	32.5	1.00	359	0	–	143.8
	200	300	260	2.3	0.88	0.46	25.0 *	34.8	1.00	359	0	–	114.3
	200	300	260	2.3	0.70	0.46	25.0 *	44.1	1.00	359	0	–	130.8
	200	300	260	2.3	1.00	0.46	25.0 *	42.4	1.00	359	0	–	162.8
Deng et al. [49] & Luo [50]	150	300	259	1.89	0	0.56	40.0	28.1	1.55	380	0.25	348	145.0
	150	300	259	1.89	0.40	0.56	40.0	26.7	1.55	380	0.25	348	136.0
	150	300	259	1.89	0.60	0.56	40.0	25.4	1.55	380	0.25	348	130.0
	150	300	259	1.89	0.80	0.56	40.0	23.5	1.55	380	0.25	348	113.0
	150	300	259	1.89	1.00	0.56	40.0	22.1	1.55	380	0.25	348	107.6
	150	300	259	1.89	0	0.43	40.0	22.6	1.55	380	0.25	348	157.0
	150	300	259	1.89	1.00	0.43	40.0	27.5	1.55	380	0.25	348	128.0
Zhang et al. [51]	151	300	263	3.00	0	0.57	20.0	24.6	2.49	357	0	–	57.0
	154	300	263	3.00	1.00	0.60	20.0	27.3	2.49	357	0	–	59.5
	153	302	265	2.50	0	0.57	20.0	24.4	2.49	357	0	–	67.5
	152	307	270	2.50	1.00	0.60	20.0	23.7	2.49	357	0	–	66.5
	152	305	268	2.00	0	0.57	20.0	31.0	2.49	357	0	–	90.0
	151	305	268	2.00	1.00	0.60	20.0	27.0	2.49	357	0	–	70.0
	152	309	272	1.50	0	0.57	20.0	28.3	2.49	357	0	–	188.5
	153	306	269	1.50	1.00	0.60	20.0	28.3	2.49	357	0	–	133.5
	153	308	271	1.00	0	0.57	20.0	24.7	2.49	357	0	–	265.0
	154	306	269	1.00	1.00	0.60	20.0	23.0	2.49	357	0	–	254.5
	153	306	269	1.50	0.30	0.60	20.0	24.7	2.49	357	0	–	168.0
	152	308	271	1.50	0.50	0.60	20.0	25.9	2.49	357	0	–	175.0
	152	307	270	1.50	0.70	0.60	20.0	26.6	2.49	357	0	–	150.0
Zhao et al. [52]	120	120	105	1.71	1.00	0.34	20.0	42.7	1.25	481	0	–	43.2
	120	180	158	1.71	1.00	0.34	20.0	42.7	1.25	459	0	–	53.0
	120	240	212	1.70	1.00	0.34	20.0	42.7	1.25	421	0	–	62.4
	120	300	266	1.69	1.00	0.34	20.0	42.7	1.25	414	0	–	88.9
Xiao & Lan [53]	150	300	258	1.50	0	0.43	25.0 *	24.8	1.96	358	0.25	560	165.0
	150	300	258	1.50	0.50	0.39	25.0 *	28.0	1.96	358	0.25	560	149.0
	150	300	258	1.50	1.00	0.37	25.0 *	27.2	1.96	358	0.25	560	137.0
Ni et al. [54]	150	300	267	1.85	0	0.50	31.5	24.8	0.90	370 **	0	–	169.0
	150	300	267	1.85	0.25	0.50	31.5	26.8	0.90	370 **	0	–	170.0
	150	300	267	1.85	0.25	0.50	31.5	26.8	0.90	370 **	0	–	156.0
	150	300	267	1.85	0.25	0.50	31.5	26.8	0.90	370 **	0	–	169.0
	150	300	267	1.85	0.50	0.50	31.5	23.7	0.90	370 **	0	–	155.0
	150	300	267	1.85	0.50	0.50	31.5	23.7	0.90	370 **	0	–	165.0
	150	300	267	1.85	0.50	0.50	31.5	23.7	0.90	370 **	0	–	160.0
	150	300	267	1.85	0.75	0.50	31.5	23.4	0.90	370 **	0	–	152.0
	150	300	267	1.85	0.75	0.50	31.5	23.4	0.90	370 **	0	–	142.0
	150	300	267	1.85	0.75	0.50	31.5	23.4	0.90	370 **	0	–	155.0
Wu et al. [55]	120	200	165	1.50	1.00	0.42	40.0	31.6	1.60	394	0.24	302	54.5
	120	200	165	1.50	0	0.42	40.0	33.2	1.60	394	0.32	302	61.5
	120	200	165	1.50	1.00	0.52	40.0	24.4	1.60	394	0.24	302	49.6
	120	200	165	1.50	0	0.52	40.0	25.0	1.60	394	0.24	302	50.0
	120	200	165	1.50	1.00	0.42	40.0	32.9	1.60	394	0.26	302	57.3
	120	200	165	1.80	1.00	0.42	40.0	33.7	1.60	394	0.32	302	57.8
	120	200	165	1.20	0	0.52	40.0	24.2	1.60	394	0.24	302	53.6
	120	200	165	1.50	1.00	0.38	40.0	36.5	1.60	394	0.24	302	57.5
	120	200	165	1.50	1.00	0.63	40.0	16.8	1.60	394	0.24	302	43.8
	120	200	165	1.50	0	0.42	40.0	33.3	1.60	394	0.24	302	54.7
	120	200	165	1.20	1.00	0.42	40.0	32.5	1.60	394	0.32	302	66.1
	120	200	165	1.50	1.00	0.42	40.0	32.6	1.60	394	0.32	302	61.1
Liao [56]	150	300	265	2.00	0	0.47	37.5	24.3	1.91	407	0.25	424	162.0
	150	300	265	1.50	0.50	0.55	37.5	23.6	1.91	407	0.25	424	182.0
	150	300	265	1.50	0.50	0.55	37.5	23.6	1.91	407	0.38	424	171.0
	150	300	265	2.00	0.50	0.55	37.5	23.6	1.91	407	0.25	424	200.0
	150	300	265	2.00	0.50	0.55	37.5	23.6	1.91	407	0.38	424	192.0
	150	300	265	2.50	0.50	0.55	37.5	23.6	1.91	407	0.25	424	160.0
	150	300	265	2.50	0.50	0.55	37.5	23.6	1.91	407	0.38	424	145.0
	150	300	265	1.50	1.00	0.53	37.5	24.7	1.91	407	0.25	424	168.0
	150	300	265	1.50	1.00	0.53	37.5	24.7	1.91	407	0.38	424	154.0
	150	300	265	2.00	1.00	0.53	37.5	24.7	1.91	407	0.25	424	149.0
	150	300	265	2.00	1.00	0.53	37.5	24.7	1.91	407	0.38	424	138.0
	150	300	265	2.50	1.00	0.53	37.5	24.7	1.91	407	0.25	424	153.0
	150	300	265	2.50	1.00	0.53	37.5	24.7	1.91	407	0.38	424	140.0

* The maximum aggregate size was not available. It was assumed to be 25 mm, which is a common size in practice. ** The strength grade of the longitudinal steel reinforcement was Q335, but the measured yield strength was not provided; it was assumed as 370 MPa per Chinese codes.

**Table A2:** Shear strength design equations.

References	Concrete’s Contribution	Stirrups’ Contribution
ACI318-2014 [61]	$v_{c}=(0.167\sqrt{f_{c}}+17\rho_{s}\frac{d}{a})$ (a/d≥2.5) $v_{c}=(3.5-2.5\frac{d}{a})(0.167\sqrt{f_{c}}+17\rho_{s}\frac{d}{a})$ (a/d<2.5)	$v_{s}=\rho_{\mathrm{sv}}f_{\mathrm{yv}}$
EN1992-1-1:2004 [62]	$\begin{array}{l} v_{c}=0.12(1+\sqrt{200/d}){\left[100\rho_{s}(f_{c}-8)\right]}^{1/3}\\ \;\;\;\;\geq 0.035{\left(1+\sqrt{200/d}\right)}^{2/3}\sqrt{f_{c}-8} \end{array}$ (d: in mm)	
GB50008-2010 [63]	$v_{c}=\frac{1.75}{a/d+1}f_{t}$ $\frac{a}{d}=\min[\max(\frac{a}{d},\;1.5),\;3.0]$	
JSCE Guideline No. 15 [64]	$\begin{array}{l} v_{c}=\min(1.5,\;\sqrt[{4}]{1000/d})\times \\ \;\;\;\;\;\;\;\min(1.5,\;\sqrt[{3}]{100\rho_{s}})\times 0.2\sqrt[{3}]{f_{c}} \end{array}$ (d: in mm)	
NZS3101-2006 [65]	$v_{c}=(0.70+0.015s_{\max})\times \min\{1.0,\;{\left(\frac{400}{d}\right)}^{0.25}\}k\sqrt{f_{c}}$ $0.08\leq k=0.07+10\rho_{s}\leq 0.20$ (d: in mm)	
Zsutty [57,58]	$v_{c}=2.175{\left(f_{c}\rho_{s}\frac{d}{a}\right)}^{\frac{1}{3}}$ (a/d≥2.5) $v_{c}=2.175(2.5\frac{d}{a}){\left(f_{c}\rho_{s}\frac{d}{a}\right)}^{\frac{1}{3}}$ (a/d<2.5)	
Simplified MCFT [66,67]	$v_{c}=\beta \sqrt{f_{c}}d_{v}/d$ $\beta =\frac{0.4}{1+1500\varepsilon_{x}}\frac{1300}{1000+s_{\mathrm{xe}}}$ $\varepsilon_{x}=\frac{V_{c}}{2E_{s}\rho_{s}bd}(1+\frac{a}{d_{v}})$ $s_{\mathrm{xe}}=d_{v}\times \min(1.0,\;\;\frac{35}{16+s_{\max}})$ $d_{v}=\max(0.9d,\;0.72h)$	
CSCT [70,71]	$v_{c}=\frac{1}{6}\frac{2}{1+120\frac{\varepsilon d}{16+s_{\max}}}\sqrt{f_{c}}$ $\varepsilon =\frac{V_{c}(a-\frac{d}{2})}{E_{s}\rho_{s}bd(d-c/3)}\frac{0.6d-c}{d-c}$ $c=d\rho_{s}\frac{E_{s}}{E_{c}}(\sqrt{1+\frac{2E_{c}}{\rho_{s}E_{s}}}-1)$	
Zhang’s Plasticity Theory [72]	$v_{c}=0.25v_{0}f_{c}(\sqrt{1+{\left(\frac{a-x}{h}\right)}^{2}}-\frac{a-x}{h})h/d$ $v_{0}=1.512\frac{15\rho_{s}+0.58}{\sqrt{f_{c}}}(1+\frac{1}{\sqrt{h/1000}})$ (h: in mm) $x\approx \{\begin{array}{c} 0.74(a-2h),\;\;\;\;\;\;\frac{a}{h}\geq 2.0\;\;\;\\ 0,\;\;\;\;\;\;\;\;\;\;\;\;\;\;\;\;\;\;\;\;\frac{a}{h}<2.0 \end{array}$	

Note: (a) Elastic modulus of steel reinforcement is taken as 200 GPa; (b) Elastic moduli of NAC and RAC are estimated as: *E*_c_ = 22,000 × (*f*_c_/10)^0.3^ and *E*_c_ = 0.71 × 22,000 × (*f*_c_/10)^0.3^, respectively [61]; (c) RAC’s tensile strength is estimated as: *f*_t_ = 0.30 × (*f*_c_ – 8)^2/3^ [73].
